# Editorial: Evidence-Based Practices to Reduce Falls and Fall-Related Injuries Among Older Adults

**DOI:** 10.3389/fpubh.2018.00222

**Published:** 2018-08-21

**Authors:** Cassandra W. Frieson, Maw P. Tan, Marcia G. Ory, Matthew Lee Smith

**Affiliations:** ^1^Fall Injury Prevention and Rehabilitation Services, Birmingham, AL, United States; ^2^Department of Medicine, University of Malaya, Kuala Lumpur, Malaysia; ^3^Center for Population Health and Aging, Texas A&M University, College Station, TX, United States; ^4^School of Public Health, Texas A&M University, College Station, TX, United States; ^5^College of Public Health, The University of Georgia, Athens, GA, United States

**Keywords:** older adults, aging, prevention, evidence-based interventions, fall-related injuries

## Tribute

This Research Topic is in memory of Dr. William “Bill” Satariano, a prolific scholar and dear friend who dedicated his career to improving the health and well-being of older adults. His scientific and practice contributions helped shape the field of healthy aging and the potential for interventions making a difference across the life-course. As a noted social epidemiologist with a concern for addressing real-world problems, Bill's research spanned many topics including cancer rehabilitation and survival, the built environment and health behaviors, and technology to promote physical activity among older adults. His work has direct implications for understanding the determinants of falls and fall-related injuries as well as the identification of multi-sectorial public health solutions.

## Overview

Falls and fall-related injuries have emerged as serious global health concerns facing older adults aged 65 years and older. Falls are known to be a leading cause of death among older adults and, when not fatal, contribute to functional limitations, mobility reductions, and loss of independence. Beyond the older adult, falls and related injuries place burden on their families and greater society in terms of caregiving and healthcare-related costs. As fall incidence rates increase alongside our growing globally aging population, fall-related mortality, hospitalizations, and costs are expected to reach never seen before heights.

Because falls occur in clinical and community settings, additional efforts are needed to understand the intrinsic and extrinsic factors that cause falls among older adults; effective strategies to reduce fall-related risk; and the role of various professionals in interventions and efforts to prevent falls (e.g., nurses, physicians, physical therapists, occupational therapists, health educators, social workers, economists, policy makers). By working together at multiple levels, we have the ability to reduce fall-related risks within respective settings, integrate and leverage risk reduction efforts across settings, and ultimately enhance policies and systems necessary for garnering support and instituting regulations to promote and finance fall prevention efforts.

Four guest co-editors have come together to address these issues from a multi-disciplinary perspective that reflects an appreciation of the clinical, community, and policy context in which falls occur. To embody this collective approach to fall prevention, encompassed within this Research Topic are 23 articles surrounding four interrelated topical areas: community-based interventions; clinical integration and intervention; special populations; and policy and systems.

## Community-based interventions

For fall prevention activities to reach the masses in communities, efforts are needed across the aging services network and public health system. This section begins with an article illustrating the importance of determining fall-related risk among community-dwelling older adults and recommending opportunities and implications for intervention in community settings (Satariano et al.). In the context of Stepping On, this section continues with a series of articles highlighting the formulation, translation, and implementation of fall prevention interventions delivered through the aging services network (Mahoney et al.; Mahoney et al.; Schlotthauer et al.). These articles are relevant and applicable to other evidence-based fall prevention interventions and emphasize the importance of these processes to formalize the programmatic elements and mechanisms for grand-scale delivery in community-based settings. One key to fall prevention in community settings is to establish a diverse and broad delivery infrastructure of trained individuals capable of reaching large numbers of older adults. One strategy to enhance the delivery infrastructure is to engage and train students (e.g., allied health, nursing, public health), who can then effectively lead programs (Der Ananian et al.). This section concludes with an examination of common intervention outcomes used in evidence-based community interventions and suggests that chronic disease self-management programs can influence fall-related self-efficacy (Graham et al.).

## Clinical integration and intervention

Given that older adults frequently interact with healthcare providers and professionals, the healthcare system is vital to creating integrated systems that complement and support community-based fall prevention efforts. This section begins with an article describing the Stopping Elderly Accidents, Deaths, and Injuries (STEADI) Toolkit, an initiative developed by the Centers for Disease Control and Prevention (CDC), and its adaptation for use among pharmacists (Karani et al.). Another article reports the efforts of 49 organizations to embed the STEADI Toolkit into clinical settings while also referring older adults to home assessments and community-based fall prevention programs (Coe et al.). To guide assessment and management of fall risk in clinical settings among older adults with previous falls, the next study reviews medical charts to determine opportunities to improve primary care practice (Phelan et al.; Phelan et al.). Two articles focus on fall prevention approaches for first responders in emergency medical systems. One study examines the feasibility and effectiveness of a program developed to enhance fall-related screening and risk identification practices of first responders (Lindgren et al.). Another tests the feasibility and effectiveness of brief on-the-scene interventions delivered by first responders to link and refer older adults to community-based fall prevention programs (Phelan et al.). To illustrate a clinical-community approach, researchers examined the effectiveness of integrating clinical activities of the Otago Education Program (OEP) in a community-based exercise program (Harnish et al.). The section concludes with an article that examines the effectiveness of modular bed absence sensors to detect bed exits among hospitalized older adults (Subermaniam et al.).

## Special populations

Often, the aim of translational efforts is to adapt or tailor interventions for use among new populations or settings as well as incorporate new delivery modalities. This section contains a collection of articles that highlight research efforts to tailor and adapt existing interventions to be more appropriate for special populations. Two translational articles surround the OEP. One illustrates the effectiveness of a delivery model using non-physical therapists, which has implications for wider intervention uptake (Shubert et al.). The other outlines the translation process and effectiveness of a model tailored for at-risk adults with intellectual and/or developmental disorders (Renfro et al.). Another feasibility study reports the potential benefits of regimented cognitive training for balance and mental health outcomes among cognitively impaired older adults (Smith-Ray et al.). Finally, this section reports qualitative findings from a series of studies among people with dementia and their caregivers to identify intervention adaptations and promising strategies for appropriate fall prevention (Meyer et al.). Collectively, these articles support the need for assessing and modifying existing interventions and strategies for diverse sets of older adults with diverse needs for fall prevention.

## Policy and systems

This section contains articles that describe the policies and systems needed to support integrated, multi-level fall prevention activities. It begins with an article from the Administration for Community Living/Administration on Aging, a national funder and supporter of community-based fall prevention programs in the United States, to describe their mission and activities to prevent falls among older adults over time (Kulinski et al.). The following two articles describe large multi-disciplinary collaboratories that strive to create systems changes for fall prevention and establish policy to support fall prevention strategies. One describes a concept for state-wide coalitions to encompass multiple topics important to healthy aging (e.g., fall prevention, chronic disease management, physical activity), given that the root causes, necessary partners, and interventions/solutions are similar and integrated (Ory et al.). The other describes an inter-disciplinary network of injury prevention task forces with state-wide reach, and a fall prevention task force that simultaneously focuses on reducing fall risk among older adults and youths (ages 0 to 4 years) (Smith et al.). This section continues with two articles from a CDC-funded multi-state, multi-level intervention that included clinic-based (i.e., STEADI Toolkit, OEP) and community-based (e.g., Stepping On, Tai Chi: Moving for Better Balance) solutions to prevent falls and fall-related injuries and deaths. One provides an account of successes, challenges, and lessons learned over this multi-year project, which can be used by other communities striving to create systems and policies to prevent falls (Shubert et al.). The other documents systems changes over time among the three funded states and their activities to leverage resources and collaborate for sustainability (Smith et al.). This section concludes with an article that utilizes asset mapping to examine the delivery of A Matter of Balance relative to fall-related emergency medical response calls to illustrate the need for strategic partnerships and planning for fall prevention dissemination in communities (Smith et al.).

## Conclusion

Falls among older adults are a multi-faceted problem that requires multi-faceted, multi-level, and integrated solutions. This Research Topic highlights that there is no “one size fits all” approach to prevent falls and that communities must leverage their strengths and partnerships to protect older adults. Communities must strive to successfully integrate efforts across aging services networks, public health systems, and healthcare to create communities with systems able to educate, screen, treat, rehabilitate, and refer older adults to ultimately reduce rates of fall-related risk, injury, and death. However, older adults are not always the primary target for fall prevention interventions. Because the list of key players and change agents is long and diverse, efforts are needed to engage professionals and organizations to ensure they can adequately address falls in their area. The establishment of formalized systems, coalitions, and task forces is needed to integrate interventions and approaches across sectors and influence policy. Further, because communities encounter challenges when attempting to reach and serve older adults in different settings, intervention contents, formats, and modalities must be translated and evolve over time. While there are multiple possible approaches to address falls in communities, Research Topics like this are essential to document the successes, challenges, and lessons learned, which can facilitate intervention replicability, expansion, and sustainability while reducing erroneous spending on ineffective approaches.

## Author contributions

All authors listed have made a substantial, direct and intellectual contribution to the work, and approved it for publication.

### Conflict of interest statement

The authors declare that the research was conducted in the absence of any commercial or financial relationships that could be construed as a potential conflict of interest.

